# The relationship between dizziness and sleep: a review of the literature

**DOI:** 10.3389/fneur.2024.1443827

**Published:** 2024-08-29

**Authors:** Roeland B. van Leeuwen, Tjard R. Schermer, Henri P. Bienfait

**Affiliations:** ^1^Apeldoorn Dizziness Centre, Gelre Hospitals, Apeldoorn, Netherlands; ^2^Department of Primary and Community Care, Radboudumc Research Institute for Medical Innovation, Radboud University Medical Center, Nijmegen, Netherlands; ^3^Department of Neurology, Gelre Hospitals, Apeldoorn, Netherlands

**Keywords:** dizziness [35, 838], sleep, review, vestibular dysfunction, benign paroxismal positional vertigo, Meniere’s disease, vestibular migraine, OSAS

## Abstract

**Background:**

The relation between vestibular disorders and (quality of) sleep is underexplored scientifically and the complex interactions between vestibular and sleep disorders are far from being well understood. Some studies have been conducted on the association between patients with vestibular disorders and (the occurrence of) sleep disorders, other studies have been published on the prevalence of dizziness complaints in patients with sleep disorders. The quality of sleep in patients with vestibular disorders generally receives little attention in clinical practice.

**Objective:**

To establish what is currently known about the mutual relationship between dizziness and sleep, and to assess whether or not there is evidence of causality with regard to this relationship.

**Methods:**

After systematically searching four literature database up until 1 April 2024, selected studies were summarized and evaluated through a (critical) review.

**Results:**

Ultimately, 42 studies were selected and evaluated. Patients with dizziness in general and patients with a specific vestibular disorder like Benign Paroxysmal Positional Vertigo, Vestibular Migraine, Meniere’s disease, and vestibular hypofunction were significantly more likely to have sleep disorders than control groups. A causal relationship is not supported due to the nature of the studies. In patients with obstructive sleep apnea syndrome there were significantly more vestibular complaints, and more disorders in the vestibular system were identified.

**Conclusion:**

Dizziness complaints often co-exist with a sleep disorder. To what extent this sleep disorder influences dizziness is not clear. Paying attention to the quality of sleep in patients with a vestibular disorder seems to be important. In patients with OSAS, consideration should be given to vestibular complaints and dysfunction.

## Introduction

Dizziness is a common complaint in primary care settings, emergency departments, and clinics. The most involved specialties in hospitals are neurology and otolaryngology surgery. Dizziness and vertigo are frequent and disabling symptoms in primary care practices but remain unexplained in 40–80% of patients (1). These symptoms may have serious individual and societal effects, causing restriction of daily activities in 40% of affected individuals (2). Falls pose an important health risk in the elderly and are known to be associated with morbidity and mortality (3). The most frequently assessed diagnoses in patients with dizziness are Benign Paroxysmal Positional Vertigo (BPPV), Vestibular Migraine (VM), Meniere’s disease (MD), and Persistent postural-perceptual dizziness (PPPD). These vestibular disorders are well treatable with maneuvres (BPPV), medication (VM), intratympanic injections (MD), and cognitive therapy (PPPD).

There are several factors that influence the quality of life in patients with dizziness, such as anxiety, depressive mood, and sleep disturbance. These factors can affect the clinical presentations and therapeutic outcomes (4). Sleep is a basic human need and is critical to both physical and mental health. More than 50 million Americans have chronic sleep disorders (5). There are several different types of sleep–wake disorders, of which insomnia is the most common (6). Other sleep–wake disorders include obstructive sleep apnea, parasomnias, narcolepsy, and restless leg syndrome.

Several socio-economic, cultural, and racial factors influence sleep duration. Short sleep duration is more common in people who are older, care for young children, are unmarried, work long hours, consume alcohol, or are overweight or obese (7). Studies have reported that sleep duration of either more or less than 7 or 8 h in a 24-h period is associated with cardiovascular disease, diabetes, obesity, depression, automobile accidents, work failures, learning and memory problems, and excess mortality (1, 8).

In recent years, the relationship between vestibular disorders and quality of sleep has received increasing attention. Conversely, studies have been performed showing more complaints of dizziness in patients with primary sleep disorders. However, the relation between vestibular disorders and (quality of) sleep is still underexplored scientifically and the complex interactions between vestibular and sleep disorders are far from being well understood. Because we think that in clinical practice the effect of sleep disorders is underestimated in patients with dizziness and vice versa, we aimed to establish what is currently known about the mutual relationship between dizziness and sleep, and to assess whether there is evidence of causality in this relationship.

## Methods

### Search strategy

We performed a literature review of published studies. PubMed, Embase, Web of Science, and Google Scholar were systematically searched for articles published from database inception to April 1, 2024. The complete search strategies including keywords are listed in Supplementary Table S1.

### Selection of articles

Articles eligible for the review were (1) primary studies based on patient level data about the relationship between vestibular disorders and quality of sleep or vice versa or (2) reviews of such studies, all written in the English language. Studies with a sample size <10 patients were excluded.

Two reviewers (RBvL and HPB) independently completed level-1 (i.e., title and abstract) screening, followed by a level-2 (full-text) screening of potentially eligible articles. A pilot exercise was conducted initially for both levels of screening to ensure consistency between the two reviewers. Discrepancies were settled through discussion with a third reviewer (TRS).

Initially, 1,112 articles were selected based on title and abstract; 42 articles were included after full-text screening. The included articles were grouped into and subsequently described and discussed in separate entities. Most articles primarily focus on the quality of sleep in patients with known vestibular diseases like BPPV, VN, MD (24 articles). A smaller number of articles (18 articles) focus on patients with primary sleep disorders, like OSAS, and assessed the association with complaints like dizziness and vertigo.

## Results

### Dizziness and sleep disorders in general (and vice versa)

Four studies have focused on the somatic complaints of patients with sleep disorders. Using data from a deidentified US medical claims database, a group of patients with untreated insomnia (*n* = 139,959) was compared with a group of subjects without insomnia (*n* = 836,975) (9). The untreated insomnia group was more likely to experience daytime fatigue, dizziness, and somnolence. These results confirmed a previous study from Korea in 12,499 patients (1, 8) which found similar results in a group of patients with sleep disorders (*n* = 864) compared with a control group (*n* = 2,340). In yet another study, the association between sleep disorders and falls was examined in older individuals from the general population while considering the influence of age and dizziness (10). Data were derived from the population-based cross-sectional Health Research in the Region of Augsburg (KORA) study, in which information was collected through standardized telephone interviews with 4,127 men and women aged over 65 years. The results of this study suggested that the positive relationship between a trend toward longer sleep duration, trouble falling and staying asleep and falls is strongest in older individuals (>75 years). The most important limitation of this study is its cross-sectional design; therefore, no causal inferences can be made regarding the association between sleep disturbances and falls.

From the perspective of patients with dizziness, four studies have been conducted to explore associations with sleep disorders. Using data from the National Health Interview Study, Albathi and Agrawal evaluated the relationship between vestibular vertigo and sleep duration in a large, representative sample of US adults (11). Vestibular vertigo and sleep duration were well-defined. Individuals with vestibular vertigo had a relative risk ratio of 1.75 for sleeping less than 6 h a day relative to regular sleep duration and an adjusted relative risk ratio of 1.55 for sleeping more than 8 h a day. In another study from Japan, 252 patients with chronic dizziness were evaluated for their quality of sleep; the prevalence of sleep disturbance (i.e., Pittsburgh Sleep Quality Index (PSQI) -J global score > 6) was 65.1% (12). Two other studies also showed a relation between dizziness and vertigo with abnormal sleep (3, 13).

Studies on dizziness and sleep in adolescents are rare. Filippopulos et al. (14) studied the potential risk factors for vertigo and dizziness in adolescents and evaluated their variability by different types of vertigo. The study population consisted of 1,482 children and adolescents between the ages of 12 and 19 years. Sleep duration was identified as an independent risk factor, next to gender, stress, muscular pain in the neck and shoulder, and migraine. A limitation of this study, similar to most cross-sectional questionnaire studies, is that reversed causation cannot be ruled out. For the above-mentioned studies, the most important limitation is their cross-sectional design. Therefore, no causal inferences can be made regarding the association between sleep disturbances and dizziness and/or falls.

### Meniere’s disease and sleep disorders

Meniere’s disease (MD) is characterized by attacks of vertigo, tinnitus, and hearing loss (15). These attacks are often accompanied by nausea and vomiting. Additionally, there is a progressive hearing loss over time. Pathologically, MD is associated with hydropic distension of the endolymphatic system, but the etiology remains unknown. The presentation of MD is highly variable, and its clinical course is characterized by acute exacerbations and spontaneous remissions (15). Studies on MD and its relation to quality of sleep are conducted in the fields of epidemiology, risk factors, quality of life, and treatment.

In the study by Bruderer et al. (16) the incidence of MD and the characteristics of MD patients compared to control patients were assessed. It was a retrospective population-based follow-up study with nested case–control analyses using data from the UK-based Clinical Practice Research Datalink. Sleeping disorders and depression were more prevalent among MD patients than among controls.

Nakayama et al. (17) evaluated the quality of sleep in MD patients using polysomnography. In this prospective study, 53 patients with active, unilateral MD refractory to medical management were included, and their results were compared with healthy controls matched for age and sex. The most important findings were decreased deep sleep and an elevated arousal index.

In a cross-sectional, case–control study on vestibular disorders and sleep disturbances, Mutli and Topcu (18) evaluated the quality of sleep in 25 patients with MD, 22 with BPPV, 21 patients with unilateral peripheral vestibular loss (UPL), 23 patients with vestibular migraine, and 43 controls. They applied the Dizziness Handicap Inventory (DHI), the Beck Depression Inventory, the PSQI, and the Limits of Stability questionnaires. The total PSQI scores of the controls were significantly better than those with MD, VM, BPPV, or UPL. This study concluded that patients with vestibular symptoms have decreased sleep quality.

A systematic review and meta-analysis looking at risk factors for MD was performed by Hu et al. (19). They included 18 cohort studies and 7 case–control studies. Results showed that age and sleep disorder were independent risk factors for MD.

In a pilot study, Nakayama et al. investigated the effect of continuous positive airway pressure (CPAP) on the manifestations of Meniere’s disease in patients with concomitant obstructive sleep apnea syndrome (20). It was a prospective study involving 20 patients with active, unilateral Meniere’s disease refractory to medical management who also had concurrent OSAS. All patients were selected to undergo CPAP. Evaluation was performed after 6 months. Audiometric testing, caloric testing, and DHI were conducted before and after starting therapy. Results showed that although caloric testing did not show significant differences, audiometric testing and results of DHI were significantly improved after CPAP therapy.

The studies mentioned above had several limitations: in some cases, they could not state with certainty whether the diagnosis of MD was confirmed by a specialist (16), no history of sleep problems was attained, and no questionnaires like the PSQI were used to assess sleep quality (17). There was a lack of polysomnography (18), absence of a control group, and no data about the frequency of vertigo attacks in the treatment period (20).

### Vestibular migraine and sleep disorders

Vestibular Migraine (VM) is a vestibular episodic disease characterized by attacks of vertigo in combination with headache. In 2012, the Bárány Society Association and the International Headache Society issued a consensus document on diagnostic criteria (21). As a relatively new vestibular central disease diagnosis, VM has received increasing interest. The pathophysiology is unknown, and there is overlap in clinical symptoms with Meniere’s disease. The relationship between sleep and migraine is well established, but analysis of sleep structure in VM is limited (22).

Wu et al. (23) studied sleep quality and sleep structure in patients with VM. In this cross-sectional case–control study, the PSQI questionnaire and polysomnography were used to compare the clinical characteristics of sleep disorders in 49 patients with VM, 52 patients with migraine, and 54 controls. Compared with the migraine and control groups, the VM group had a higher incidence of poor sleep quality and a greater severity of poor sleep quality. The VM group showed reduced sleep efficiency and reduced proportions of REM and slow-wave sleep compared to the other two groups.

In a prospective cohort of patients with VM, Liu et al. (24) evaluated the severity of VM attacks and contributing factors. Risk factors for more severe attacks included PSQI score, duration of illness, and time of onset. Therefore, appropriate management of sleeping problems may reduce the severity of attacks in VM patients.

Zhou et al. (25) studied the cyclic alternating pattern (CAP) in non-rapid eye movement (REM) sleep in patients with VM. In their cross-sectional study, they included 35 VM patients, 35 migraine patients, and 30 controls. Data were collected using the PSQI, HADS, and PSG. The vestibular migraine group had a higher CAP time, index, sequences, and A2% + A3%. The authors concluded that VM patients have a hypersympathetic function, a more active awakening system, and disordered sleep structure.

The effects of lifestyle modification on VM were studied by Roberts et al. (26) in a prospective within-participants repeated-measures study. Participants were 28 adults with definite VM who were willing to be managed without pharmacological intervention. They were instructed to improve restful sleep, exercise, eat at regular mealtimes, and avoid dietary triggers. Participants who reported a larger increase in restful sleep were more likely to report improvement in both dizziness and headache.

All of the above studies have limitations: small sample size of groups (23, 25) and no control group (24, 26).

### BPPV and sleep disorders

Benign paroxysmal positional vertigo (BPPV) is a short-term, sudden-onset, peripheral vestibular disease triggered by angular changes in head position relative to gravity. This means that activities such as lying down in bed, getting in or out of bed, turning right or left in bed, or bending the head can trigger rotational dizziness and positional nystagmus. BPPV is typically categorized as canalithiasis or cupulolithiasis. It is the most common cause of dizziness, with a lifetime prevalence of 2.4%. While the cause is often unknown, secondary causes of BPPV can include trauma, Meniere’s disease, vestibular neuronitis, and migraine. BPPV also increases the risk of falling, especially in the elderly. Fortunately, BPPV can be effectively treated with canalith repositioning maneuvres.

Studies in the field of BPPV and sleep disorders can be divided into two groups. One group focuses on examining the quality of sleep in patients with BPPV, while the other group investigates the role of sleep as a risk factor for BPPV recurrence.

Studies focused on the quality of sleep in patients with BPPV included comparisons between BPPV patients and control groups (27–29). All studies utilized at least the PSQI, with some also conducting polysomnography. Across these studies, BPPV patients consistently reported significantly worse sleep quality compared to healthy controls. However, it is important to note that these studies were all cross-sectional, and thus, causality cannot be inferred from their findings.

Another group of studies investigated the factors for the recurrence of BPPV through literature reviews (30, 31). Sfakianaki et al. (31) reviewed 30 studies involving 13,358 patients to identify risk factors for BPPV recurrence, with sleep disorders emerging as one of many potential risk factors. However, this review noted limitations such as a high degree of heterogeneity among the studies and a predominance of retrospective designs. Therefore, sleep disorders may be underestimated as risk factors for recurrence of BPPV and require further investigation.

Li et al. (30) conducted a systematic review and meta-analysis of 24 articles to assess risk factors for BPPV recurrence. While many risk factors were evaluated, sleep disorders were not isolated as a specific risk factor for BPPV recurrence. Limitations of this review include a high degree of heterogeneity among the studies and varying levels of publication quality.

In summary, patients with BPPV often experience a decline in sleep quality. However, the evidence does not conclusively support sleep disorders as an evidence-based risk factor for BPPV recurrence.

### Unilateral and bilateral vestibular hypofunction and sleep disorders

Few studies have been reported on quality of sleep in patients with vestibular hypofunction. Causes for unilateral and bilateral hypofunction can be diverse, like vestibular neuritis, Meniere’s disease, or gentamicin intoxication. Most patients with vestibular hypofunction complain about balance problems. Stress may be an important factor for compensation and rehabilitation in patients with vestibular hypofunction (32). In addition, balance in general may be influenced by physical fatigue (33).

One study evaluated sleep behavior and its relationship to otoneurologic parameters in a group of patients with chronic unilateral vestibular hypofunction without self-reported sleep disturbances, with healthy subjects serving as a control group (34). The patients with vestibular hypofunction were found to be spending less time sleeping. A moderate correlation was found between longer disease duration and reduced sleep time. The authors offer no explanation for this correlation.

Another study assessed the impact of sleep quality on the balance and quality of life of individuals with vestibular hypofunction (35). If patients had a combination of vestibular hypofunction and sleep disorders, there was a higher risk of falls.

### Vestibular disorders and obstructive sleep apnoea syndrome

Obstructive sleep apnea syndrome (OSAS) is the most common sleep disorder, characterized by recurrent episodes of upper respiratory tract obstruction resulting in breath cessation (apnea) or reduction of airflow (hypopnea) during sleep. Symptoms often include heavy snoring and daytime sleepiness (36). From a clinical perspective, the long-term effects on cortical–subcortical functions due to intermittent hypoxemia, associated with the severity of OSAS, are not yet completely understood. However, it is known that sleep fragmentation and sleep deprivation may lead to impairments in memory, executive functioning, attention, and motor coordination.

Studies about the relationship between vestibular disorders and OSAS can be divided in 1. Incidence or prevalence of OSAS in patients with a vestibular disorder, 2. Vestibular symptoms in patients with OSAS, 3. Vestibular functions in patients with OSAS, and 4. Effect of treatment of OSAS on vestibular complains.

### Incidence/prevalence of OSAS in patients with vestibular symptoms

Three studies have specifically examined the incidence or prevalence of OSAS in patients presenting with vestibular symptoms (37–39). Maas et al. (38) conducted a cross-sectional study to determine the prevalence of high-risk OSAS in patients reporting dizziness. They included 704 Dutch adult patients who completed the DHI and the Stop-BANG questionnaire. About 20% of the patients were found to be at high risk of OSAS based on the Stop-BANG questionnaire. However, this study did not include PSG to confirm the OSAS diagnosis, which is a notable limitation.

Sowerby et al. (39) investigated the association between sleep apnea, daytime somnolence, and chronic idiopathic dizziness in a case–control study. They compared 46 patients with idiopathic dizziness (ID), 20 positive controls with benign paroxysmal positional vertigo (BPPV), and 69 negative controls with hearing loss but no dizziness. Although the ID group exhibited significantly more daytime somnolence and higher Multivariable Apnea Risk test scores compared to the other groups, PSG was not performed in this study, limiting the ability to confirm the OSAS diagnosis.

Finally, Bery et al. (37) aimed to determine the frequency of OSAS in patients with persistent postural-perceptual dizziness (PPPD). In a small group of 25 patients with PPPD, 56% were diagnosed with OSAS via PSG. However, the lack of a control group in this study is a notable limitation.

### Vestibular symptoms in patients with OSAS

Two population-based studies by Tsai et al. (40) and Byun et al. (41) examined the risk of vertigo in patients with OSAS using control groups of non-OSAS patients. Both studies concluded that sleep apnea is an independent risk factor for vertigo. However, it is important to note that these studies were retrospective in nature and thus did not define the term vertigo specifically.

Chen et al. (42) performed a study to investigate the relationship between vertigo and OSAS. They divided patients with OSAS, confirmed by PSG, into a subgroup with and a subgroup without vertigo. Among the OSAS patients with vertigo, 41.3% had REM-related OSAS, whereas only 15.9% of OSAS patients without vertigo exhibited REM-related OSAS. However, this study was also retrospective and did not provide a specific definition of vertigo.

Another study by Foster and Machala (43) investigated the clinical descriptions of dizziness, vestibular diagnoses, and treatment responses in OSAS patients with dizziness. They found that out of 52 OSAS patients who were dizzy and received treatment, 19 responded positively with the disappearance of their dizziness after treatment. These patients typically experienced repeated spells of non-positional vertigo, resembling vestibular paroxysmia. This study raises the possibility of a new vestibular entity related to OSAS, but its retrospective nature is a notable limitation.

Overall, while these studies provide valuable insights into the relationship between OSAS and vertigo, their retrospective designs and lack of clear definitions limit the ability to establish causality and warrant further prospective investigations.

### OSAS and vestibular and postural functions

Vestibular function tests, including vestibular evoked myogenic potentials (VEMP), caloric testing, video head impulse test (vHIT), and posturography, have been employed in several studies to assess vestibular function in patients with OSAS.

Four studies (44–47) investigated VEMP responses in OSAS patients compared to controls. All these studies demonstrated significant differences in o-VEMP and c-VEMP responses between OSAS patients and controls.

Caloric testing was evaluated in three studies (46, 48, 49), all of which found significantly higher rates of canal paresis in OSAS patients compared to controls. There was also a positive correlation between the DHI and canal paresis.

Three studies (44, 47, 50) investigated vHIT in OSAS patients, all showing significantly different pathological vestibulo-ocular reflex (VOR) gains compared to controls, except for one study (47).

Posturography was assessed in a study by Micarelli et al. (50), which included 32 severe OSAS patients and 32 healthy persons. The OSAS patients exhibited significant impairments in classical posturography parameters compared to controls.

### Effect of OSAS treatment on vestibular complaints

Lastly, Alessandrini et al. (51) evaluated the effect of continuous positive airway pressure (CPAP) treatment on postural and vestibular functions in OSAS patients. They found that postural instability and dizziness-related conditions may improve after 12 months of CPAP treatment, although VOR gain did not significantly improve.

Overall, these studies demonstrate alterations in vestibular function in OSAS patients and suggest potential improvements with CPAP treatment, highlighting the importance of addressing vestibular symptoms in OSAS management.

## Discussion

This review of studies regarding the interrelationship between sleep and dizziness, and vice versa, provides us with a deeper understanding of the relationship, particularly highlighting its complexity. In summary, it is important to realize that sleep disorders occur more frequently in patients with dizziness, and conversely, patients with sleep disorders are more likely to experience dizziness. Due to the often small cohorts or the retrospective nature of the studies, little can be said about causality. Among specific groups with vestibular disorders such as Meniere’s disease, vestibular migraine, BPPV, or unilateral vestibular loss, sleep disorders were more prevalent than in control groups. However, the available studies do not demonstrate causality here either.

Obstructive Sleep Apnea Syndrome (OSAS), a commonly occurring condition, is generally associated with significant morbidity, such as hypertension, vascular incidents, and cognitive decline. Our review indicates that multiple studies have shown that the vestibular system can be affected by OSAS. Among the many complaints that OSAS patients present, dizziness occurs more frequently than in control groups. However, a clear relationship between the occurrence of OSAS and (unexplained) dizziness could not be established. Sleep centers should be aware that when diagnosing OSAS, inquiries about vestibular symptoms such as dizziness and balance issues should also be made.

**Figure 1:**
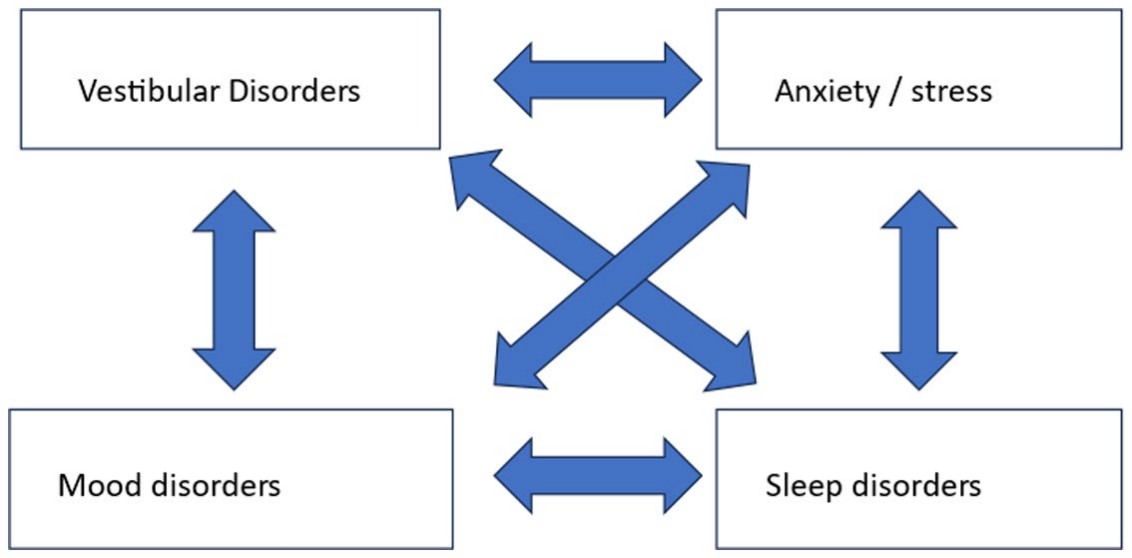
Interrelationship between vestibular disorder, anxiety, mood disorder and sleep.

In patients with dizziness, we are generally aware that stress and mood can play a significant role in the somatic complaint of dizziness (52). Stress and mood can be the primary cause of dizziness, but they can also be caused by a primary vestibular disorder. Sleep disorders should also be considered alongside these accompanying complaints. Because stress, mood, and sleep are intertwined, a multifactorial influence arises when it comes to their relation with dizziness, as shown in Figure 1.

So far, we are often inclined to inquire about stress and mood when managing patients with dizziness, but the quality of sleep is usually not thoroughly assessed. For stress and mood, we can use the Hospital Anxiety and Depression Scale (HADS), and for sleep evaluation, for example, the Pittsburgh Sleep Quality Index (PSQI).

The major issue is to consider what do we do when we identify a sleep disorder in a patient with dizziness? The same applies to accompanying stress, depression and/or mood disorders. For instance, we encounter a male or female patient with vestibular migraine, experiencing weekly dizziness attacks, who wishes to be treated. Let us suppose this patient has significant insomnia, based on an extensive questionnaire. Do we treat the patient solely for his or her vestibular migraine, with preventative medication, or do we first address their insomnia, or both simultaneously? Lifestyle interventions are mentioned in some studies, but robust research to establish their effects on quality of life is lacking. Therefore, the answer as to whether or not to implement any treatments requires further well-designed intervention studies. And this further research may be worthwhile: if it would show that primarily treating a sleep disorder is sufficient to achieve a significant reduction in dizziness attacks then we may avoid treatment with preventive medication in the case of vestibular migraine or injections in Meniere’s disease.

So, what do all these studies mean for clinical practice? The fact that sleep disorders are more prevalent in vestibular disorders necessitates the evaluation of sleep quality in patients with dizziness. In our view, sleep should be assessed in the same way as stress and psychiatric disorders are. Despite the lack of demonstrated causality, our suggestion is to also treat accompanying sleep disorders. Additionally, research should focus on a possible causal link between the severity of vestibular symptoms and sleep quality.

## Conclusion

This literature review shows that sleep disorders are more common in patients with vestibular disorders, and patients with a sleep disorder are more likely to experience dizziness. A clear causal link has yet to be demonstrated, but it is suspected. Obtaining information about sleep seems important, as is information about stress and mood. More research should be conducted on the importance, particularly in terms of timing, of treating a concurrent sleep disorder in patients with dizziness. Until we have this data, we propose to treat a concurrent sleep disorder synchronously with the treatment of the vestibular disorder, all in consultation with the patient.
