# Supplementary material for: The relationship between dizziness and sleep: a review of the literature
Source: Front Neurol. 2024 Aug 29;15:1443827. doi: 10.3389/fneur.2024.1443827 (PMC11390376; doi:10.3389/fneur.2024.1443827)
Supplement: Supplementary file 1 [file Table_1.DOCX]

**Table S1**. Search terms used in the respective literature databases

| **Pubmed** |
| --- |
| (("Dyssomnias"[Mesh] OR "Sleep Wake Disorders"[Mesh] OR "Sleep Disorders"[tiab] OR "Sleep Disorder"[tiab] OR dyssomnia[tiab] OR dyssomnias[tiab] OR "sleep wake disorder"[tiab] OR "sleep wake disorders"[tiab] OR "sleep deprivation"[tiab] OR parasomnias[tiab] OR parasomnias[tiab] OR "Sleep Initiation and Maintenance Disorders"[Mesh] OR "Sleep Initiation and Maintenance Disorders"[tiab] OR "Sleep Initiation and Maintenance Disorder"[tiab] OR "Disorders of Excessive Somnolence"[Mesh] OR "Idiopathic Hypersomnia"[Mesh] OR "Sleep Disorders, Intrinsic"[Mesh] OR "Sleep Apnea Syndromes"[Mesh] OR "Sleep Disorders, Circadian Rhythm"[tiab] OR "Circadian Rhythm Sleep Disorders"[tiab] OR "Sleep Disorders, Circadian Rhythm"[Mesh] OR "Parasomnias"[Mesh] OR "REM Sleep Parasomnias"[Mesh] OR "Disorders of Excessive Somnolence"[tiab] OR "Idiopathic Hypersomnia"[tiab] OR "Sleep Disorders, Intrinsic"[tiab] OR "Sleep Apnea Syndromes"[tiab] OR "Intrinsic Sleep Disorders"[tiab] OR "Disorder of Excessive Somnolence"[tiab] OR "Sleep Apnea Syndrome"[tiab] OR "Intrinsic Sleep Disorder"[tiab] OR Parasomnia*[tiab] OR "Narcolepsy"[tiab] OR "Narcolepsy"[Mesh] OR "Sleep Apnea, Obstructive"[Mesh] OR "Sleep Apnea, Obstructive"[tiab] OR "Obstructive Sleep Apnea"[tiab] OR "Obstructive Sleep Apneas"[tiab] OR OSAS[tiab] OR SAS[tiab] OR CSAS[tiab] OR "Sleep Apnea, Central"[Mesh] OR "Sleep Apnea, Central"[tiab] OR "Central Sleep Apnea"[tiab] OR "Central Sleep Apneas"[tiab] OR "Sleep Hygiene"[Mesh] OR "Sleep Hygiene"[tiab] OR "Sleephygiene"[tiab])) AND (("Dizziness"[Mesh] OR Dizziness[tiab] OR "Vertigo"[Mesh] OR Vertigo[tiab] OR Dizzyness[tiab] OR bppv[tiab] OR benign paroxysmal positional vertigo[tiab] OR Vertigos[tiab])) |
| **Embase** |
| ('benign paroxysmal positional vertigo'/exp OR 'benign paroxysmal positional  vertigo':ab,ti OR 'bppv':ab,ti OR 'meniere disease'/exp OR meniere*:ab,ti OR  'vestibular migraine'/exp OR 'vestibular migraine':ab,ti OR 'vestibular neuronitis'/exp  OR 'vestibular neuronitis':ab,ti OR 'vestibular neuritis':ab,ti OR 'vestibular  paroxysmia'/exp OR 'vestibular paroxysmia':ab,ti OR 'bilateral vestibulopathy'/exp OR  'bilateral vestibulopathy':ab,ti OR 'bilateral vestibular hypofunction':ab,ti OR  'dizziness'/exp OR 'dizziness':ab,ti OR 'vertigo'/exp OR  'vertigo':ab,ti) AND  ('sleep'/exp OR 'sleep disorder'/exp OR ‘sleep*’:ab,ti OR 'insomnia'/exp OR  'insomnia':ab,ti) |
| **Web of Science** |
| (TS=benign paroxysmal positional vertigo OR TS=bppv OR TS=meniere* OR  TS=vestibular migraine OR TS=vestibular neuronitis OR TS=vestibular neuritis OR  TS=vestibular paroxysmia OR TS=bilateral vestibulopathy OR  TS=bilateral vestibular hypofunction OR TS=dizziness OR TS=vertigo)  AND  (TS=sleep* OR TS=insomnia) |
| **Google Scholar** |
| (bppv OR "benign paroxysmal positional vertigo" OR meniere* OR (vestibular  AND (migraine OR neuritis OR paroxysmia)) OR "bilateral vestibular hypofunction" OR Dizziness OR Vertigo) AND (Sleep* OR insomnia*) |
